# Supplementary material for: Splice-Junction-Based Mapping of Alternative Isoforms in the Human Proteome
Source: Cell Rep. Author manuscript; Available in PMC 2020 Jan 15. (PMC6961840; doi:10.1016/j.celrep.2019.11.026)
Supplement: 2 [file NIHMS1546469-supplement-2.zip › DF1/PXD010154/Lung/Lung_35_AKT1_MLPTYPHSR.pdf]

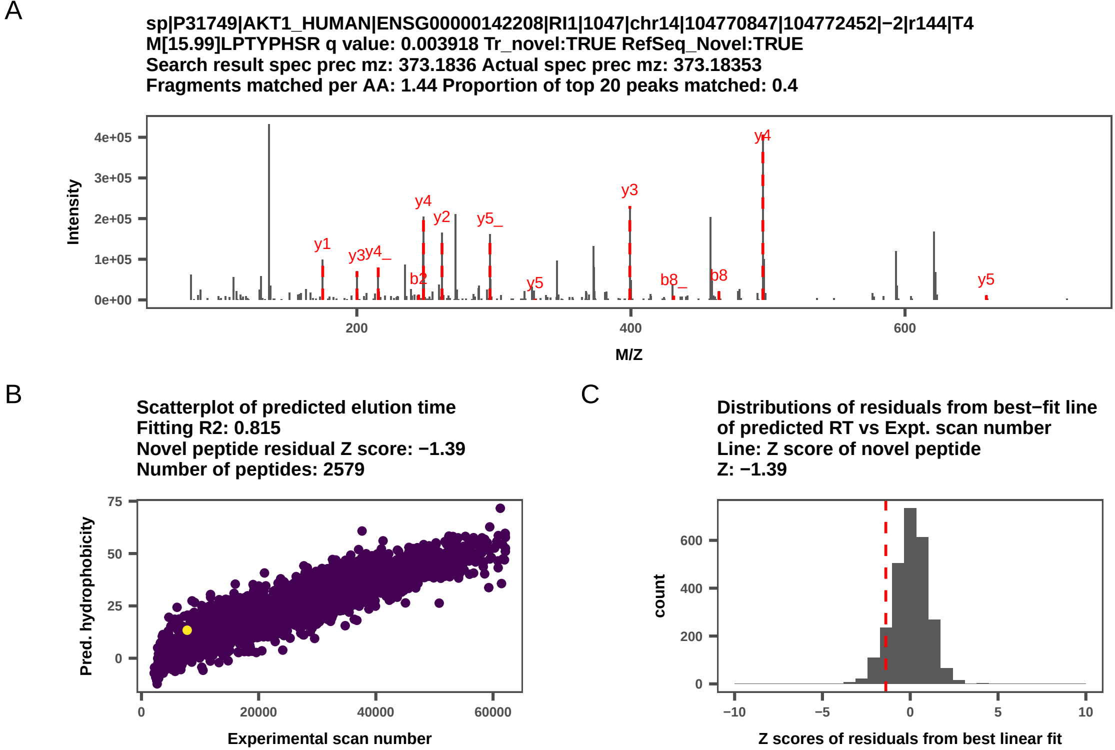
A
sp|P31749|AKT1_HUMAN|ENSG00000142208|RI1|1047|chr14|104770847|104772452|−2|r144|T4
M[15.99]LPTYPHSR q value: 0.003918 Tr_novel:TRUE RefSeq_Novel:TRUE
Search result spec prec mz: 373.1836 Actual spec prec mz: 373.18353
Fragments matched per AA: 1.44 Proportion of top 20 peaks matched: 0.4
Intensity
4e+05
3e+05
2e+05
1e+05
0e+00
y4
y3
y4
y2
y5_
y1
y3y4_
b2
y5
b8_
b8
y5
200
400
600
M/Z
B
Scatterplot of predicted elution time
Fitting R2: 0.815
Novel peptide residual Z score: −1.39
Number of peptides: 2579
Pred. hydrophobicity
75
50
25
0
0
20000
40000
60000
Experimental scan number
C
Distributions of residuals from best−fit line
of predicted RT vs Expt. scan number
Line: Z score of novel peptide
Z: −1.39
count
600
400
200
0
−10
−5
0
5
10
Z scores of residuals from best linear fit
